# Low Dose Decitabine Treatment Induces CD80 Expression in Cancer Cells and Stimulates Tumor Specific Cytotoxic T Lymphocyte Responses

**DOI:** 10.1371/journal.pone.0062924

**Published:** 2013-05-09

**Authors:** Li-Xin Wang, Zhen-Yang Mei, Ji-Hao Zhou, Yu-Shi Yao, Yong-Hui Li, Yi-Han Xu, Jing-Xin Li, Xiao-Ning Gao, Min-Hang Zhou, Meng-Meng Jiang, Li Gao, Yi Ding, Xue-Chun Lu, Jin-Long Shi, Xu-Feng Luo, Jia Wang, Li-Li Wang, Chunfeng Qu, Xue-Feng Bai, Li Yu

**Affiliations:** 1 Department of Hematology, Chinese PLA General Hospital, Beijing, China; 2 Department of Hematology, Navy General Hospital, Beijing, China; 3 Department of Pathology and Comprehensive Cancer Center, The Ohio State University Medical Center, Columbus, Ohio, United States of America; 4 State Key Laboratory of Molecular Oncology, Cancer Institute/Hospital, Chinese Academy of Medical Sciences, Beijing, China; Istituto Superiore di Sanità, Italy

## Abstract

Lack of immunogenicity of cancer cells has been considered a major reason for their failure in induction of a tumor specific T cell response. In this paper, we present evidence that decitabine (DAC), a DNA methylation inhibitor that is currently used for the treatment of myelodysplastic syndrome (MDS), acute myeloid leukemia (AML) and other malignant neoplasms, is capable of eliciting an anti-tumor cytotoxic T lymphocyte (CTL) response in mouse EL4 tumor model. C57BL/6 mice with established EL4 tumors were treated with DAC (1.0 mg/kg body weight) once daily for 5 days. We found that DAC treatment resulted in infiltration of IFN-γ producing T lymphocytes into tumors and caused tumor rejection. Depletion of CD8^+^, but not CD4^+^ T cells resumed tumor growth. DAC-induced CTL response appeared to be elicited by the induction of CD80 expression on tumor cells. Epigenetic evidence suggests that DAC induces CD80 expression in EL4 cells via demethylation of CpG dinucleotide sites in the promoter of CD80 gene. In addition, we also showed that a transient, low-dose DAC treatment can induce CD80 gene expression in a variety of human cancer cells. This study provides the first evidence that epigenetic modulation can induce the expression of a major T cell co-stimulatory molecule on cancer cells, which can overcome immune tolerance, and induce an efficient anti-tumor CTL response. The results have important implications in designing DAC-based cancer immunotherapy.

## Introduction

A major challenge in cancer immunotherapy is immune evasion by cancer cells [1]. During tumor development and progression, tumors build up an immune suppressive network, including tumor associated myeloid cells and various regulatory T cells [2], [3]. Cancer cells themselves are genetically unstable; they can down-regulate major histocompatibility complex (MHC) class I molecules [4], [5] and lose the expression of tumor antigens [6], [7], [8]. In addition, cancer cells do not normally express key co-stimulatory molecules such as CD80, but rather express some co-inhibitory molecules that render tumor antigen specific T cell tolerance [9]. All these factors prevent the induction of an efficient T cell response to tumors. Thus, overcoming immune evasion is of great importance in cancer immunotherapy.

Epigenetic evidence suggests that in cancer cells, some key immune stimulatory molecules are regulated by DNA methylation in their promoter region. Some well known tumor antigens such as cancer testis antigens (CTAs) are almost exclusively regulated by DNA methylation [10], [11], [12], [13], [14], [15]. MHC class I and its antigen presentation machinery have also been shown to be regulated by DNA methylation [16], [17], [18], [19]. In addition to CTAs and MHC molecules, there is also evidence that adhesion molecules [16], [20] such as ICAM-1 and LFA-3, and the co-stimulatory molecules [19], [20] such as CD40 and CD86 can be regulated by DNA methylation in cancer cells. Thus, demethylating agents that can upregulate expression of tumor antigens, MHC class I, and adhesion/co-stimulatory molecules in cancer cells should be useful in enhancing tumor immunogenicity and their susceptibility to immune destruction. Indeed, there is a body of evidence that suggests demethylation treatment can dramatically increase cancer cell susceptibility to destruction by T cells [11], [15], [17], [21]. However, there is no direct *in vivo* evidence that demethylation treatment of cancer leads to a specific anti-tumor T cell response.

Decitabine (DAC), a DNA demethylating agent [22], has recently emerged as a potent therapeutic for the treatment of pre-leukemic hematological disease-MDS [23], [24], established leukemia [25], [26], [27] and advanced lung cancer [28]. Low dose DAC can cause sustained anti-tumor effects even after discontinuation of treatment [24], [29], [30], suggesting that an active immune response may be induced in the treated patients. To determine whether DAC treatment can induce anti-tumor immune responses *in vivo*, we studied the effect of low dose DAC treatment in mice with established T cell lymphoma EL4 tumors. We found that transient and low dose DAC treatment resulted in the induction of an antitumor cytotoxic T lymphocyte (CTL) response that mediated tumor regression. DAC-induced CTL response appears to be elicited by the induction of CD80 expression on EL4 cells. In addition, we also found that a transient, low-dose DAC treatment can induce CD80 gene expression in a variety of human cancer cells. This study provides the first evidence that epigenetic modulation can induce the expression of a major T cell co-stimulatory molecule on cancer cells, which can overcome immune tolerance, and induce an efficient anti-tumor CTL response.

## Methods

### Mice

C57BL/6 and CBF1 mice were purchased from Beijing Vital-River Lab Animal Technology Co. Ltd. C57BL/6 congenic strain mice B6.SJL-Ptprc^a^ Pepc^b^/Boy-M (CD45.1^+^) were provided by Dr. Chunfeng Qu (State Key Laboratory of Molecular Oncology, Cancer Institute/Hospital, Chinese Academy of Medical Sciences, Beijing, China). All mice were maintained at Chinese PLA General Hospital (CPGH) in specific pathogen-free conditions. All animal experiments were performed in accordance with national and institutional guidelines for animal care and were approved by the animal use and care committee, CPGH.

### Cell Culture and EL4 Subclone Establishment

All cell lines were originally obtained from American Type Culture Collection (ATCC). EL4 cells (mouse T cell lymphoma/leukemia cell line) were cultured and maintained in Dulbecco’s Modified Eagle Medium (DMEM) supplemented with 10% heat-inactivated equine serum (Hyclone), 100 µg/ml penicillin/streptomycin and L-glutamine. The cells were incubated at 37°C in 5% CO_2_. EL4 subclones were established using limiting dilution in 96-well round-bottomed tissue culture plates based on CD80 expression. Two EL4 subclones, i.e. EL4 C45 with high expression of CD80, and EL4 C3 with no expression of CD80, were used for this study. The human leukemia cell lines K562 (cell line derived from a patient with chronic myeloid leukemia at blast crisis), U937 (acute myelogenous leukemia, M5), THP-1 (acute myelogenous leukemia, M5), NB4 (acute myelogenous leukemia, M3), Kasumi-1 (acute myelogenous leukemia, M2), Molt-4 (T-cell acute lymphoblastic leukemia), Hut-78 (T cell lymphoma) and Raji (Burkitt’s lymphoma) were grown and maintained in RPMI-1640 (Hyclone) medium supplemented with 10% fetal bovine serum.

### In vitro and in vivo Treatment with Decitabine

Decitabine (DAC, Xian-Janssen pharmaceuticals Ltd, China) was dissolved in phosphate-buffered saline (PBS) (pH 7.4) to obtain 100 µM stocks and stored at −20°C. For *in vitro* studies, DAC was added to cell culture medium to a final concentration of 0.25 µM for 72 hours. The same concentration of Cytidine (Sigma) in PBS or PBS only was added to cells as control treatment. 24 hours after treatment cells were harvested for further study. For *in vivo* studies using DAC, mice with established EL4 tumors were injected with DAC (1.0 mg/kg body weight in 200 µl PBS) or PBS i.p. once daily for 5 consecutive days. Mice were sacrificed 7–10 days after completion of drug treatment and the tumors excised were processed for tumor infiltrating lymphocytes (TIL) analysis.

### Reverse Transcription-PCR (RT-PCR)

Total RNA was extracted from DAC-treated or vehicle-treated EL4 cells and other human leukemia and lymphoma cells using TRIzol reagent (Invitrogen) according to manufacturer’s instruction. RT was performed using Reverse Transcription System (Promega) on 1 µg of total RNA, and PCR amplifications were then performed using primers shown in Table 1.Simultaneous amplification of glyceraldehyde-3-phosphate dehydrogenase (GAPDH) gene using primers for mouse (forward 5′-GATGCCCCCATGTTTGT-3′; reverse 5′-CCGTTCAGCTCTGGGATGA -3′) and humans (forward 5′-GAGTCAACGGATTTGGTCGT-3′; reverse 5′-TTGATTTTGGAGGGATCTCG-3′) was used as an internal control for the amount and integrity of the RNA analyzed. All PCR products were resolved on agarose gels and visualized using ethidium bromide staining.

**Table 1:** PCR primers used in this study.

Genes	GenBank code	Primers	Product length
mouse CD80	NM_009855.2	5′-AGTCGTCGTCATCGTTGTCA-3′ (forward)	170 bp
		5′-CCATGTATCCCACATGGACA-3′ (reverse)	
human CD80	NM_005191.3	5′-CATCCAAGTGTCCATACCTC-3′ (forward)	805 bp
		5′-CTCTCATTCCTCCTTCTCTC-3′ (reverse)	
mouse P1A	NM_011635.1	5′-CGGAATTCTGTGCCATGTCTGATAACAAGAAA-3′ (forward)	728 bp
		5′-CGTCTAGATTGCAACTGCATGCCTAAGGTGAG-3′ (reverse)	
mouse Mela	BC113756.1	5′-CAGAGACTACCGCCGATTACA-3′ (forward)	131 bp
		5′-GGCTCCCTGTGGATCGTTTG-3′ (reverse)	
mouse Magea4	NM_020280.2	5′-TCGGAGCCAAAGGGAGTTAGA-3′ (forward)	154 bp
		5′-GGCTAGTATCACAAGGGGAGAG-3′ (reverse)	
mouse CD79b	NM_008339.2	5′-CGAGGTTTGCAGCCAAAAAG-3′ (forward)	137 bp
		5′-CACAATGCGTCCCTCTTCTG-3′ (reverse)	
mouse CD74	NM_001042605.1	5′-CCGCCTAGACAAGCTGACC-3′ (forward)	84 bp
		5′-ACAGGTTTGGCAGATTTCGGA-3′ (reverse)	
mouse CD48	NM_007649.4	5′-TGGTCCTGGAACTGCTACTG-3′ (forward)	112 bp
		5′-GGGTCCTTATGGATTTTCAGGG-3′ (reverse)	
mouse CD300a	NM_170758.3	5′-TGAGTGCCAGTATGTGAATTTGC-3′ (forward)	257 bp
		5′-ACAGGTAAAGGTCAGAGAGTCC-3′ (reverse)	
mouse CD3eap	NM_145822.2	5′- ACCCTCCCGTTTCTCCTTG-3′ (forward)	143 bp
		5′-CCAGTTTGCCCTTAACAGTCTTG-3′ (reverse)	
mouse CD274	NM_021893.3	5′-GCTCCAAAGGACTTGTACGTG-3′ (forward)	238 bp
		5′-TGATCTGAAGGGCAGCATTTC-3′ (reverse)	
mouse CD247	NM_001113394.2	5′-GGGAGGCAAACAGAGGAGG-3′ (forward)	266 bp
		5′-CTGGGAGGCTAAGAGGCTTC-3′ (reverse)	
mouse CD180	NM_008533.2	5′-GCTCCAAAGGACTTGTACGTG-3′ (forward)	143 bp
		5′-TGATCTGAAGGGCAGCATTTC-3′ (reverse)	

### Real Time PCR

Real-time PCR was performed using an ABI 7900-HT sequence system (PEApplied Biosystems, Carlsbad, CA, USA) using previously determined conditions [31]. SYBR Premix Ex Taq PCR kit (Takara Bio, Inc.) and the same primers used for RT-PCR were employed for amplifying mouse P1A, Mela, Magea4, CD80, CD79b, CD74, CD48, CD300a, CD3eap, CD274, CD247, CD180 and GAPDH genes. Each sample was assayed in triplicate, and the experiments were repeated twice. The relative amount of mRNA was calculated by plotting the Ct (cycle number), and the average relative expression for each group was determined using the comparative method (2^−△△Ct^).

### Tumorigenesis

C57BL6 mice were injected with various doses (most often using 1×10^4^ cells/mouse, s.c.) of DAC-treated or control EL4 cells. Tumor volumes were measured as ab^2^/2 (a = length; b = width) every two to three days. To induce mouse leukemia, mice were injected with 2×10^4^ EL4 cells/mouse i.v.

### In vivo CD8+ and CD4+ T Cell Depletion

C57BL/6 mice were injected with 400 µg of anti-CD4 (clone GK 1.5; BioXcell, NH, USA) or anti-CD8 (clone 53–6.72; BioXcell, NH, USA) monoclonal antibodies i.p. every 4 days immediately after treatment with DAC. The control mice were treated with purified rat IgG2b or IgG2a, respectively (BioXcell, NH, USA). The depletion effects were assessed at the endpoint of the experiment by staining of spleen and tumor cells for CD4 and CD8 using anti-CD4 (RM4.4) and anti-CD8 (5H10-1) mAbs (eBioscience), followed by flow cytometric analysis.

### Antibodies and Flow Cytometric Analysis

All antibodies used were purchased from BD Biosciences (San Diego, CA) or eBioscience (San Diego, CA). For staining of cell surface markers, cells (cancer cells, splenocytes and single cell suspensions of tumors) were stained with antibodies (FITC-, PE-, APC- or Percp- labeled CD80, CD4, CD8a, CD3, NK1.1, IFN-γ, and isotype control antibodies) in staining buffer (PBS with 1% FCS) on ice for 30 min. Cells were fixed in 1% paraformaldehyde in PBS. For detection of intracellular cytokines, cells were stimulated *in vitro* with PMA (100 ng/ml) and ionomycin (1000 ng/ml) for 5 h. Golgi^Stop^ (BD Pharmingen, USA ) was added (1/1500) during the last 2 h of incubation. The cells were first stained for the cell surface markers such as CD4 or CD8, followed by a standard intracellular cytokine staining procedure for IFN-γ. Cells were collected using a FACSCalibur® flow cytometer and data were analyzed using the FlowJo software (Tree Star, Inc., OR).

### Gene Expression Microarray Analysis

Total RNA was extracted from DAC-treated and vehicle-treated EL4 cells using TRIzol reagent (Invitrogen) according to manufacturer’s instruction. cDNA labeled with a fluorescent dye (Cy5 and Cy3-dCTP) was generated using CapitalBio cRNA Amplification and Labeling Kit (CapitalBio, Beijing, China). SmartArray (CapitalBio, Beijing, China) chips containing about 25000 mouse genes were hybridized with labeled cDNA probes on a GeneChip system. Arrays were scanned with a confocal LuxScan™ scanner and the images obtained were then analyzed using LuxScan™ 3.0 software (Both from CapitalBio, Beijing, China). Significance Analysis of Microarrays (SAM) was performed to determine differentially expressed genes.

### Bisulfite Sequencing

The methylation status of the CpG dinucleotides within two regions (oligo 1: nucleotide −792 to −335, oligo2: nucleotide −151 to +258), relative to the 5′ end of the CD80 gene, was analyzed. Genomic DNA was prepared from EL4 subclones EL4 C3 (CD80^−^) and EL4 C45 (CD80^+^), vehicle or DAC-treated EL4 cells using the Wizard Genomic DNA Purification Kit (Promega Inc, USA). For subcloning and sequencing of individual alleles, bisulfite-treated genomic DNA was amplified using the Epitect bisulfit kit (Qiagen, Germany) using the following primer pairs: fragment 1(forward 5′-GGGTATTTTTTTAAAAGAAGAGA-3′; reverse 5′-AATCCTACAAAAACATCAATCAAC-3′), fragment 2 (forward 5′- GGTTGGGTGGGAATTATTTTATT-3′; reverse 5′-ACTTAAACACCTCCTAAACTCACA-3′).

The PCR products were gel purified and cloned into the pGEM-T vector (Promega Inc, USA). The inserted PCR fragments of individual clones were sequenced using an ABI PRISMDNA sequencer (Applied Biosystems, USA).

### Mixed Lymphocyte Reaction and CD80 Blockade

T cells harvested from spleen and lymph nodes of EL4-immunized BALB/c mice were used as responder cells. DAC or vehicle-treated EL4 cells irradiated with X-ray (14 Gy) were used as stimulator cells. Responder cells and stimulator cells were incubated in 96-well plates in a ratio of 8∶1, 4∶1, 2∶1 and 1∶1, and cultured at 37°C at 5% CO_2_ for 6 days. T-cell proliferation was assessed with Cell Counting Kit-8 (Dojindo, Kumomoto, Japan) according to the manufacturer’s instructions. Briefly, after incubation for 6 days at 37°C, 10 µL water soluble tetrazolium salt (WST)-8 was added to each well, and the cells were incubated for another 4 to 6 hours at 37°C. The absorbance of the sample at 450 nm was measured. For CD80 blockade, anti-CD80 (16-10A1, eBioscience, San Diego, CA) or a control isotype matched IgG, (eBioscience, San Diego, CA) were added into co-culture wells at a final concentration of 5 µg/ml.

### IL-2 and IFN-γ ELISA

Supernatants of mixed cultures were harvested 72 h after co-culture and were analyzed for IL-2 and IFN-γ using ELISA kits (eBiosciences, San Diego, CA).

### Statistics

Statistical analyses were conducted using GraphPad Prism Software (GraphPad Software, Inc., USA). Student’s t test was used to compare tumor volume differences between two groups. For comparison of mouse survival, the Kaplan-Meier survival analysis and log-rank test were used. A *P* value less than 0.05 was considered significant.

## Results

### DAC Treatment Inhibits EL4 Cell Tumorigenesis and Leads to Regression of Established Tumors in C57BL/6 Mice

**Figure 1:**
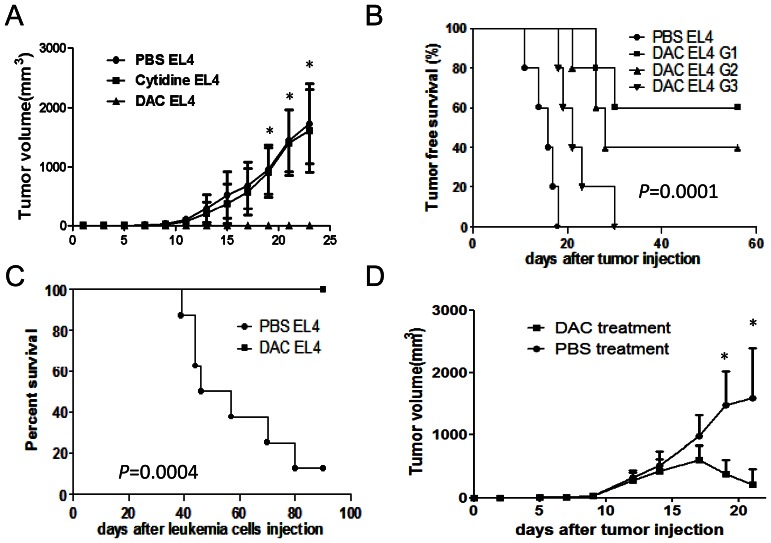
Impacts of DAC treatment on EL4 tumorigenicity and tumor growth. (A) EL4 cells were treated with DAC (0.25 µM in culture medium) or Cytidine or PBS for 72 h. Cells were then injected into each mouse s.c. at a dose of 1×10^4^ cells/mouse. Tumor size was measured in two directions every two or three days. Tumor volume was calculated using a formula: volume = (L x W^2^)/2 where L = length; W = width. *P<0.05 when DAC-treatment is compared to PBS or Cytidine treatment. Five to six mice were used in each group and data were pooled from two experiments. (B) EL4 cells treated with DAC were injected into C57BL/6 mice s.c. at a dose of 1×10^4^ cells/mouse (G1), 8×10^4^ cells/mouse (G2) or 16×10^4^ cells/mouse (G3). PBS-treated EL4 cells (1×10^4^ cells/mouse) served as control. Mouse survival data is shown. Five mice were included in each group and data shown are representative of three independent experiments. (C) DAC-treated or PBS-treated EL4 cells were injected into each mouse i.v. at a dose of 1×10^4^ cells/mouse. Mouse survival was monitored up to 100 days after tumor cell injection. Ten mice per group were used, and data shown are representative of three independent experiments. (D) Mice with established EL4 tumors were treated with DAC or PBS i.p. for 5 consecutive days starting on day 10. Data shown are representative of three experiments. Asterisks indicate statistical significance of *P*<0.05.

To test whether DAC treatment affects tumorigenicity of cancer cells in mice, EL4 cells were treated with either DAC (0.25 µM) or Cytidine (0.25 µM) or vehicle (PBS) for 72 hours. We included Cytidine as a control agent since DAC is the analogue of Cytidine. In addition, a previous study has revealed that nucleotide (Thymidine) treatment is toxic to EL4 cells [32]. Viable EL4 cells from the above mentioned treatments were then injected into C57BL/6 mice subcutaneously (s.c.) at a number of 1×10^4^ cells/mouse. At this number, Cytidine and vehicle-treated EL4 cells formed tumors in all injected mice and grew progressively; in contrast, no tumors were formed in mice receiving DAC-treated EL4 cells (Figure 1A). EL4 tumors were established in C57BL/6 mice only when very high numbers of DAC-treated EL4 cells were used (8–16 fold higher doses) (Figure 1B). Intravenous injection of vehicle-treated EL4 cells (1×10^4^ cells/mouse) led to the development of EL4-induced leukemia in CBF1 mice and caused death of most mice; in contrast, no mice receiving this number of DAC-treated EL4 cells died of leukemia (Figure 1C). To test whether *in* *vivo* injection of DAC could inhibit the growth of established tumors, EL4 cells were injected into C57BL/6 mice s.c. When tumors grew to a size of about 5×6 mm, these mice were treated with DAC or PBS i.p. daily for 5 consecutive days. In contrast to the progressive tumor growth in vehicle treated mice, DAC treatment caused continuous tumor regression even after DAC treatment was stopped (Figure 1D). Thus, DAC-treated EL4 cells show reduced capacity in tumorigenesis, and transient DAC treatment of mice with established tumors leads to continuous tumor regression.

### DAC Treatment Induces Anti-tumor CTL Responses

**Figure 2:**
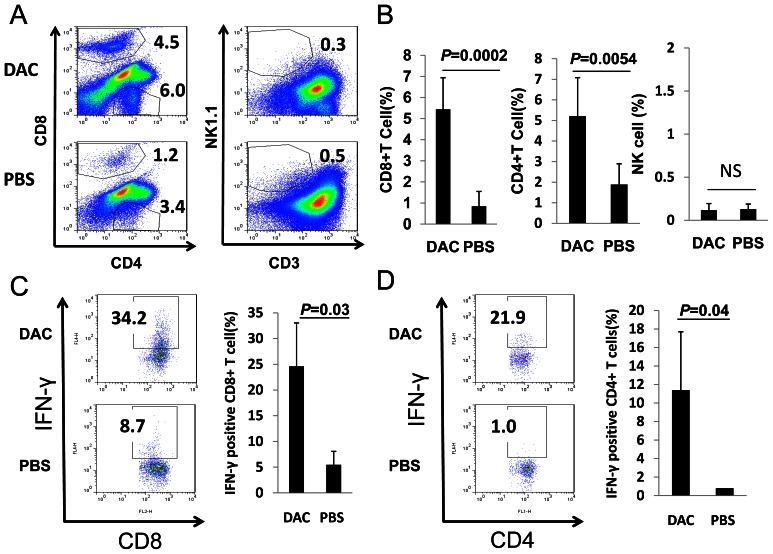
DAC treatment induces T cell infiltration into tumors. (A–B) C57BL/6 mice with established EL4 tumors were treated with DAC (1 mg/kg body weight) or PBS once daily for 5 consecutive days. 7–10 days after treatment, mice were sacrificed, tumors were harvested, and disassociated tumor cells were stained for the expression of different cell surface markers, followed by flow cytometry quantification for CD8^+^, CD4^+^ and NK1.1^+^CD3^−^ cells. (C) Flow cytometric analysis and quantification of intracellular IFN-γ production by tumor infiltrating CD8^+^ T cells. Bars represent mean+SD; n = 3–7 mice per group. (D) Flow cytometric analysis of intracellular IFN-γ production by tumor infiltrating CD4^+^ T cells. Bars represent mean+SD; n = 3–7 mice per group. Student’s t test was used for the statistical analysis. Numbers in flow cytometric figures indicate % positive cells corresponding to each gate.

**Figure 3:**
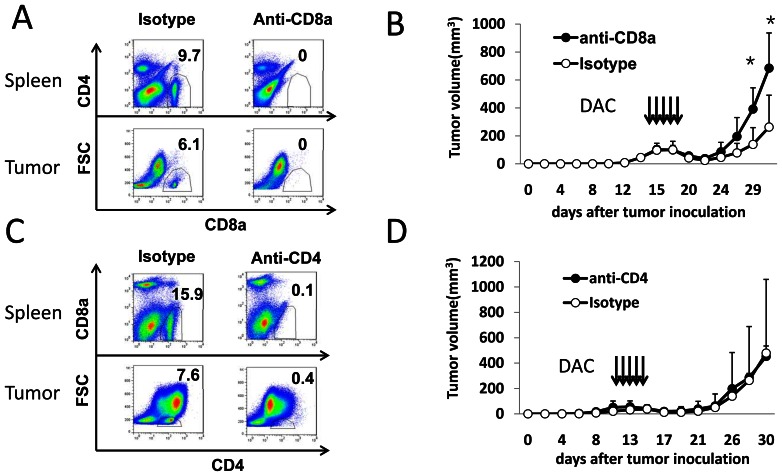
DAC-treatment leads to CD8+ T cell dependent tumor rejection. Four doses of anti-CD8 or anti-CD4 antibody (400 µg/per mouse, i.p.) were injected at 4 day intervals beginning on day 1 after DAC treatment. Three mice per group were used for the experiment shown. (A) CD8^+^ T cells in spleens and tumors were analyzed by flow cytometry after anti-CD8 antibody or an isotype-matched control antibody treatment. (B) Tumor growth in mice treated with anti-CD8 or an isotype-matched control mAb following DAC administration. Data is representative of two independent experiments with similar results. Asterisks indicate statistical significance of *P*<0.05. (C) CD4^+^ T cells in spleens and tumors were analyzed by flow cytometry after anti-CD4 antibody or an isotype-matched control antibody treatment. (D) Tumor growth of mice treated with anti-CD4 or an isotype-matched control mAb following DAC administration. Data is representative of two independent experiments with similar results. Numbers in flow cytometric figures indicate % positive cells corresponding to each gate.

The fact that the transient DAC treatment resulted in persistent tumor regression suggests that DAC treatment could have induced anti-tumor immune responses. To test this possibility, C57BL/6 mice with established EL4 tumors were treated with DAC as described above (Figure 1D). Seven to ten days later, mice were sacrificed, and mononuclear cells from tumors were stained for CD4, CD8 and NK1.1/CD3, followed by analysis using flow cytometry. As shown in Figure 2A, tumors from DAC-treated mice had significantly increased numbers of CD8^+^ and CD4^+^ T cells compared with tumors from vehicle-treated mice; NK1.1^+^ CD3^−^ NK cells were barely detectable in tumors from DAC-treated and PBS-treated mice (Figure 2A, B). The tumors from DAC-treated mice contained higher numbers of IFN-γ producing CD8+ T cells compared with tumors from vehicle-treated mice (Figure 2C, D). To determine whether increased T cell responses were responsible for DAC-induced tumor regression, C57BL/6 mice with established EL4 tumors treated with DAC were also treated with anti-CD8 or anti-CD4 antibodies or their relative control antibodies i.p. Anti-CD8 or anti-CD4 treatment resulted in complete depletion of CD8^+^ or CD4^+^ cells from spleens and tumors (Figure 3A, C). Depletion of CD8^+^ T cells (Figure 3B), but not CD4^+^ T cells (Figure 3D), resulted in more aggressive tumor growth in otherwise protected mice. Thus, DAC treatment of mice with established tumors induced CD8^+^ T cell-mediated anti-tumor immunity.

### DAC Treatment Induces CD80 Expression in Tumor Cells

**Figure 4:**
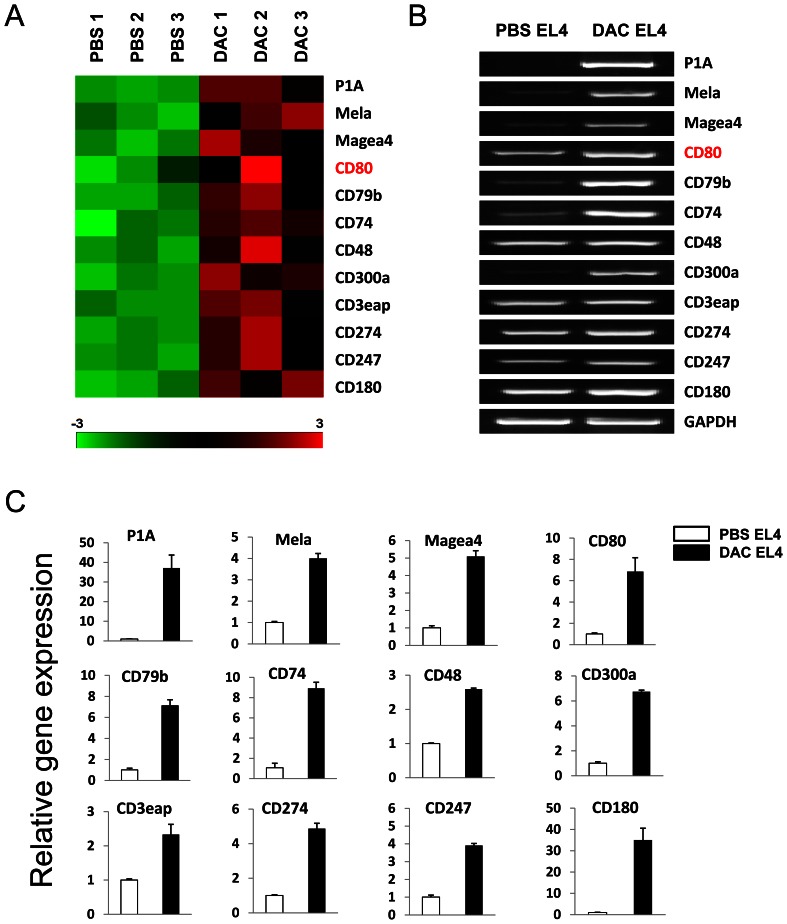
Up-regulated gene expression in DAC treated EL4 cells. The mouse SmartArray chips were hybridized with RNA derived from DAC or PBS treated EL4 cells. (A) Heatmap of some upregulated genes by DAC treatment. Columns represent microarray data obtained from 3 independent biological replicates. (B) RT-PCR was used to validate the up-regulated genes. (C) qRT-PCR was used to quantify up-regulated genes in DAC and PBS treated EL4 cells.

Since DAC treatment induced CTL responses in EL4 tumors, we hypothesized that DAC treatment can up-regulate immune stimulating molecules present on EL4 cells, which can stimulate CTL responses in immunocompetent mice. Earlier studies have reported up-regulation of MHC class molecules and tumor antigens by demethylating agents [11], [17]. However, we failed to detect upregulation of MHC class I and class II molecules in DAC-treated EL4 cells (data not shown). To further explore the mechanisms of DAC-induced tumor immunity, we compared the differential expression of genes in DAC-treated EL4 cells and controls using cDNA microarray analyses (GEO accession # GSE38473) and found that DAC treatment significantly altered the gene expression profiling in EL4 cells compared with vehicle treatment (Figure S1A). A total of 847 upregulated genes were identified in DAC-treated EL4 cells using the SAM analysis (Figure S1B). Among the upregulated genes in DAC-treated EL4 cells, 3 cancer testis antigens and 9 cluster of differentiation (CD) molecules including CD80 (B7-1), an essential T cell co-stimulatory molecule, were identified (Figure 4A). RT-PCR and qRT-PCR also verified their up-regulation (Figure 4B and 4C).

**Figure 5:**
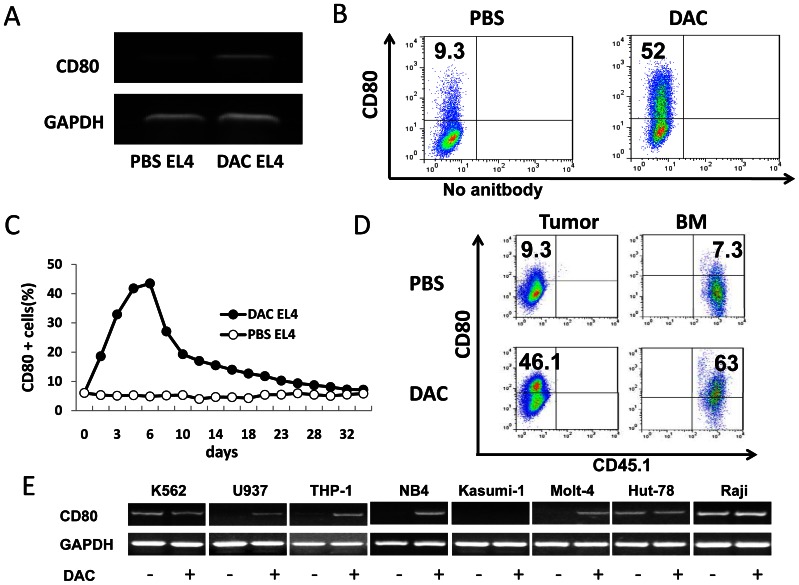
Characterization of DAC-induced CD80 gene expression in cancer cells. (A) EL4 cells were treated with DAC (0.25 µM) or PBS for 72 h. RT-PCR was used to detect CD80 gene expression in DAC and PBS treated EL4 cells. The GAPDH gene was simultaneously amplified as a loading control. (B) Flow cytometric analysis of CD80 expression in DAC-treated and PBS- treated EL4 cells. (C) EL4 cells were treated with DAC and PBS for 3 consecutive days and were maintained in the culture. Cells were harvested every two days and stained with anti-CD80 followed by flow cytometric analysis. (D) EL4 cells (CD45.2) were injected s.c. into C57BL/6 mice or i.v. into CD45.1 congenic C57BL/6 mice. DAC was administrated i.p. 2 weeks later for 5 consecutive days. EL4 cells from disassociated tumors (left panel) and bone marrows (BM) (right panel) were analyzed for the CD80 expression by flow cytometry. (E) Induction of CD80 expression in human cancer cell lines. Cancer cells were treated with DAC (0.25 µM) or PBS for 72 h. RT-PCR was used to determine CD80 gene expression. Numbers in flow cytometric figures indicate % positive cells corresponding to each gate.

To determine the persistence and stability of DAC- induced CD80 expression, EL4 cells were cultured in medium containing DAC (0.25 µM) or PBS for 72 hours. DAC-induced CD80 expression in EL4 cells was verified at both mRNA (Figure 5A) and protein level (Figure 5B). Kinetic analysis revealed that CD80 expression peaked at 3–5 days after DAC treatment, and was maintained at significant levels for about 3 weeks and then declined to the base level (Figure 5C). To test whether DAC treatment can induce CD80 expression *in vivo,* EL4 cells (CD45.2^+^) were injected s.c. into C57BL/6 mice or i.v. into CD45.1 congenic mice. Two weeks later, mice were treated with DAC (1mg/kg body weight, i.p.) for 5 consecutive days. Seven to 10 days later tumor cells from DAC- and vehicle-treated mice were examined for CD80 expression. DAC treatment significantly up-regulated CD80 expression in EL4 cells from s.c. tumors (Figure 5D, left panel) as well as in EL4 cells from bone marrows (Figure 5D, right panel). To determine whether DAC-induced upregulation of CD80 is a general phenomenon, 6 human leukemia cell lines and 2 human lymphoma cell lines were treated with DAC (1 µM for 72 hours) followed by RT-PCR analysis. Four out of 5 CD80-negative cell lines (U937, THP-1, NB4 and Molt-4) showed DAC-induced CD80 expression, while in three cell lines with pre-existing CD80 gene expression (K562, Hut-78 and Raji), the induction effect was not obvious (Figure 5E).

### DAC Induces Demethylation of the Promoter Region in CD80 Gene

**Figure 6:**
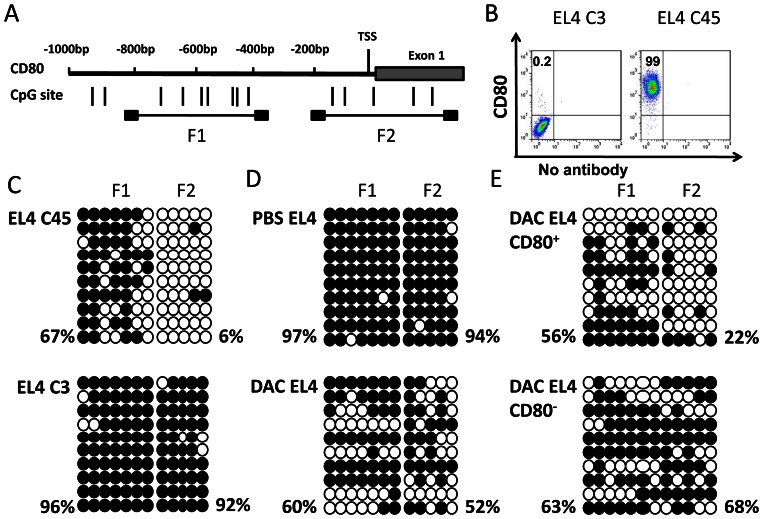
DNA methylation of CD80 promoter region in EL4 and its variant cells. (A) The distribution of CpG dinucleotides in a region 1 kb upstream of Exon 1 of CD80 gene. Numbers refer to position relative to the 5′ end of CD80. Two CpG enriched regions (F1 and F2) were selected for Bisulfite sequencing analysis. (B) Flow cytometric analysis of CD80 expression on two EL4 variants (EL4C3 and EL4C45). Numbers in flow cytometric figures indicate % positive cells corresponding to each gate. (C) Bisulfite sequencing of the CD80 promoter region in EL4C45 (upper panel) and EL4C3 (lower panel). Open and filled circles represent demethylated and methylated CpG sites, respectively. The methylation frequency at CpG sites of each cell line is indicated. (D) Bisulfite sequencing analysis of CD80 promoter region in DAC- and PBS-treated EL4 cells. (E) EL4 cells were first treated with DAC or PBS, CD80^+^ and CD80^−^ EL4 cells were then sorted by flow cytometry-based sorting. Bisulfite sequencing analysis was performed on DAC-treated, CD80^+^ and CD80^−^ EL4 cells.

To determine the underlying mechanism by which DAC induces CD80 expression in EL4 cells, we analyzed the sequence of the promoter region of murine CD80. There were no typical CpG islands in the promoter region of CD80. However, 14 CpG dinucleotides were found in Exon1 and its upstream 800 bp region (Figure 6A). Two pairs of primers were designed for bisulfite sequencing analysis of two fragments covering nucleotide −792 to −335 (F1), and nucleotide −151 to +258 (F2). We first compared the methylation status of the CpG dinucleotides in the two fragments from two EL4 subclones with or without natural expression of CD80 (Figure 6B). In F1, 96% of CpG dinucleotides were methylated in CD80^−^ cells versus 67% in CD80^+^ cells. In F2, 92% of CpG dinucleotides were methylated in CD80^−^ cells compared to 6% in CD80^+^ cells (Figure 6C). DAC treatment of EL4 cells caused a significant increase in demethylation sites in both F1 and F2 regions (Figure 6D). Among DAC-treated EL4 cells, CD80^+^ cells exhibited more demethylation sites compared with DAC-treated CD80^−^ cells (Figure 6E). Thus, CD80 gene expression in EL4 cells is closely associated with the demethylation status of its promoter region and DAC treatment significantly increases demethylation of CD80 promotor.

### DAC-induced CD80 Expression in EL4 Cells Triggers Anti-tumor CTL Response

**Figure 7:**
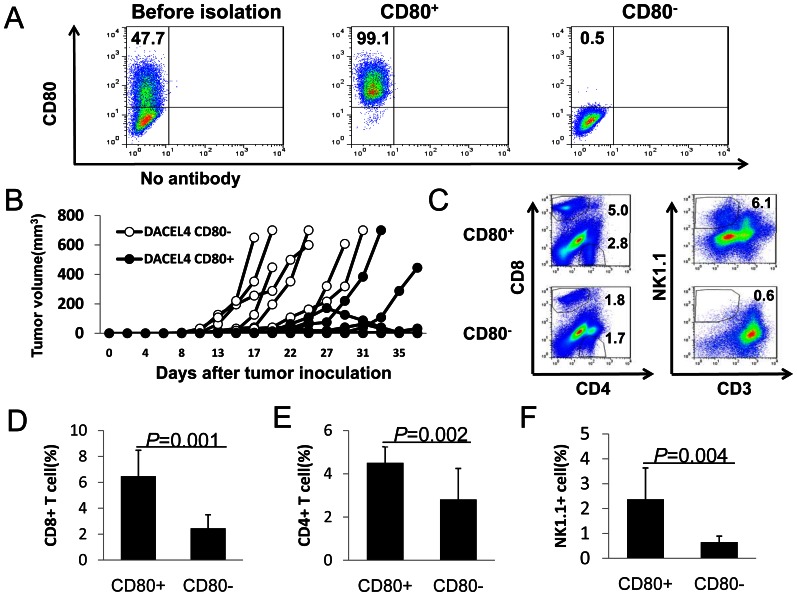
DAC educated CD80 positive, but not CD80 negative EL4 cells induce T cell responses. (A) EL4 cells were treated with DAC. CD80^+^ and CD80^−^ EL4 cells were separated by flow cytometry-based sorting. Flow cytometric analysis of CD80 expression in EL4 cells before and after sorting is shown. (B) Tumor growth kinetics of DAC-treated CD80^+^ and CD80^−^ EL4 cells. 2×10^4^ CD80^+^ or CD80^−^ DAC-treated EL4 cells were injected s.c. into each mouse. Tumor growth in individual mouse is shown. (C) Flow cytometry analysis of T cell and NK cell infiltration in CD80^+^ and CD80^−^ tumors. Single cell suspensions of tumors were stained for CD4, CD8, CD3, NK1.1 followed by flow cytometry analysis. Percentage of CD8^+^ (D), CD4^+^ (E) and NK1.1^+^CD3^−^ (F) in CD80^+^ tumors (n = 6) and CD80^−^ tumors (n = 6) were quantified. Data shown are mean+SEM and are representative of four independent experiments with similar results. Numbers in flow cytometric figures indicate % positive cells corresponding to each gate.

To determine whether DAC-induced CD80 expression in EL4 cells triggers anti-tumor CTL responses, EL4 cells were co-cultured with DAC for 72 h. CD80^+^ and CD80^−^ EL4 cells were then separated using flow cytometry-based high speed sorting to obtain highly purified CD80^+^ and CD80^−^ EL4 cells (Figure 7A). These cells were subsequently injected into C57BL/6 mice (2×10^4^ cells/mouse s.c.). All mice that received CD80^−^ EL4 cells grew tumors, while majority (60–80%) of mice that received DAC-treated CD80^+^ EL4 cells failed to grow tumor. In some cases, CD80^+^ EL4 tumors grew transiently or grew later (Figure 7B). CD80^+^ EL4 tumors contained much higher numbers of CD8^+^ and CD4^+^ T cells (Figure 7C–E) compared with CD80^−^ EL4 tumors. NK1.1^+^CD3^−^ NK cells were only detected in some cases of CD80^+^ EL4 tumors but not in CD80^−^ EL4 tumors (Figure 7C, F). Thus, DAC-induced CD80 expression triggers anti-tumor immunity *in vivo*.

**Figure 8:**
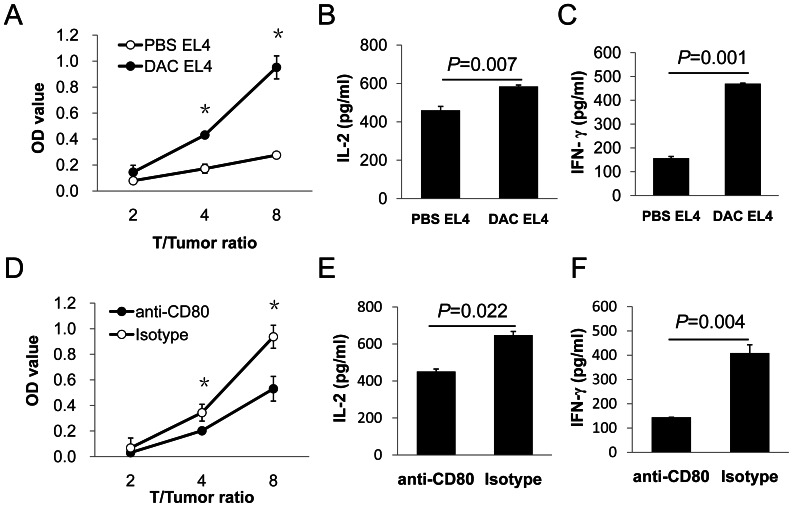
Role of DAC induced CD80 expression in stimulation of T cells. Six million irradiated EL4 cells were injected i.p. into each BALB/c mouse twice with a week interval between injections. Spleen and lymph node cells from EL4 immunized BALB/C mice were co-cultured with DAC-treated or PBS-treated EL4 cells for 6 days. (A) T cell proliferation was assessed using Cell Counting Kit-8 (CCK8, Dojindo, Kumomoto, Japan) on day 6. IL-2 (B) and IFN-γ (C) production in the culture supernatants were detected by ELISA. For CD80 blocking, spleen and lymph node cells from EL4-immunized mice were co-cultured with DAC-treated EL4 cells in the presence of 5 µg/ml anti-CD80 antibody or an isotype matched control antibody. (D) T cell proliferation was assessed using the CCK8 assay. Supernatants of co-culture were harvested 48 hours later for detection of IL-2 (E) and IFN-γ (F) by ELISA. Student’s t-test was used for statistical analysis. Data shown are representative of three experiments with similar results.

To further understand the impacts of DAC-induced tumor cell expression of CD80 on T cell activation and effector functions, we performed mixed lymphocyte culture and CD80 blockade experiments. DAC-treated EL4 cells induced stronger alloreactive T cell proliferation (Figure 8A), and more IL-2 (Figure 8B) and IFN-γ (Figure 8C) production compared with control EL4 cells. CD80 blockade significantly reduced DAC-EL4 cell-induced T cell proliferation (Figure 8D) and cytokine production (Figure 8E, F). Thus, DAC-induced CD80 expression on tumor cells can directly enhance T cell activation and effector functions.

## Discussion

In this study we show that DAC, a DNA methylation inhibitor that is currently used for the treatment of MDS, AML and other malignant neoplasms, is capable of eliciting an anti-tumor CTL response in mouse EL4 tumor model. A transient, low dose DAC exposure to EL4 cells *in vitro* and *in vivo* induces CD80 expression on cancer cells, which is likely the major cause of the induction of CTL response. Mechanistically, we show that DAC induces CD80 expression in EL4 cells via demethylation of CpG dinucleotide sites in the CD80 gene promoter.

Previous studies have revealed that demethylating agents can restore cancer cell expression of MHC class I and its antigen presentation machinery [16], [17], [18], [19], and induce the expression of tumor antigens [10], [11], thereby increasing their susceptibility to destruction by immune effector cells such as CTL. Some studies have also shown that demethylation treatment up-regulates co-stimulatory molecules such as CD40 and CD86 [19], [20]. However, it has not been shown that such a treatment can induce CD80 expression in cancer cells. In addition, there is no direct *in vivo* evidence that demethylation treatment of cancer leads to a specific anti-tumor T cell response. Thus, this study provides the first evidence that epigenetic modulation of cancer cells leads to the expression of a major T cell co-stimulatory pathway in cancer cells that triggers anti-tumor CTL responses.

In this study we found that DAC treatment induced a potent CTL response characterized by tumor infiltration of high numbers of IFN-γ producing CD8^+^ T cells. Although we also observed increased numbers of CD4^+^ T cells in tumors, depletion of CD8^+^ T cells, but not CD4^+^ T cells resulted in accelerated tumor recurrence. Thus, DAC-induced CTL responses, but not CD4^+^ T cell responses are mainly responsible for the tumor regression. Although tumor infiltrating NK cells were not induced in tumors from DAC-treated animals, increased NK cells were detected in tumors from mice injected with DAC-induced CD80^+^ EL4 cells, suggesting that higher levels of CD80 expression on tumor cells may be required for the induction of NK response in tumors.

Decitabine was shown to up-regulate the expression of genes involved in growth control and apoptosis, and down-regulate genes related to the malignant phenotype of cancer cells [33]. Microarray analysis indicates that DAC induces profound gene expression alteration in cancer cells. The up-regulated genes include those encoding for cancer testis antigens such as P1A and MAGE-A1 and many cell surface molecules such as CD80. It is thus likely that many mechanisms, such as elevated tumor antigen expression may also contribute to tumor regression. However, a few lines of evidence suggest that up-regulation of CD80 is the single most important factor that contribute to the T cell responses and tumor regression. First, CD80 is the most potent co-stimulatory molecule [34]. Classic tumor immunology studies [35], [36], [37] have revealed that ectopic expression of CD80 on tumor cells has potent effects on the induction of anti-tumor immune responses, mainly anti-tumor CTL response [35], [36], [37] and sometimes NK response [38]. In addition, tumor expression of CD80 also enhances CTL effector functions and facilitates better tumor destruction [39], [40]. Second, in this study we found that in DAC-treated tumor bearing animals, tumor cells strongly up-regulated CD80. Third, DAC-induced CD80^+^ tumor cells, but not CD80^−^ tumor cells induced potent anti-tumor CTL responses. Finally, in vitro CD80 blockade experiments suggest that blocking DAC-induced CD80 interaction with T cells directly enhance T cell proliferation and production of cytokines.

CD80 expression is normally tightly controlled, and epigenetic mechanism for the regulation of CD80 expression remains unclear. The promoter region of CD80 gene does not contain a typical CpG island. However, we found that DAC treatment induced dramatic demethylation in the CpG dinucleotides in EL4 cells. Notably, demethylation of the F2 region (nucleotide −151 to +258) appears to have a more close correlation with the induction of CD80 expression. DAC- induced CD80 expression is also observed in a number of human leukemia and lymphoma cell lines. However, those human cell lines show differential susceptibility, thus suggesting that different cancer cells may have differential expression of epigenetic modulating machinery. Further studies are required to determine this issue.

A transient low dose DAC treatment in mice with EL4 tumors resulting in the induction of tumor specific CTL response has important clinical relevance. Recently, compiling evidence suggests that low dose of demethylating agents can cause sustained anti-tumor effects even after termination of the agent used. For instance, patients with hematological cancer receiving DAC treatment often experience prolonged responses [24], [29]. Low dose azacitidine was used to treat patients with advanced non-small cell lung cancer that failed chemotherapy, very impressive responses were achieved [28]. In an animal study transient low doses of DAC exert durable antitumor “memory response” [30]. Thus, it is likely that immune responses are involved in “sustained effects” due to low dose DAC treatment in patients with cancer. Further investigation of the underlie mechanisms will reveal new therapeutic strategies of those incurable cancers.

**Figure 9:**
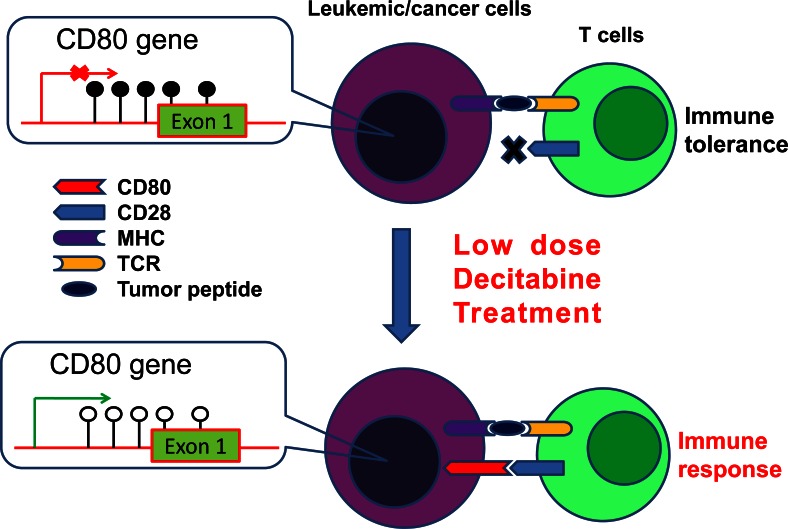
The model of DAC action in T cell response. DNA hypermethylation in the promoter and exon 1 region of CD80 gene repress the expression of CD80 in leukemic/cancer cells. Lack of co-stimulatory signal leads to T cell tolerance. Low dose decitabine treatment can restore cancer cell expression of CD80 by demethylation of the CD80 promoter region in leukemic/cancer cells. The expression of CD80 on leukemic/cancer cells can overcome T cell tolerance and lead to activation of T cells. MHC = Major histocompatibility complex; TCR = T cell receptor.

Taken together, we have demonstrated that transient, low dose DAC treatment in mice with established tumors induces anti-tumor CTL response and induction of a major co-stimulation pathway. Based on these observations, we propose a mechanistic model depicting the mechanisms of DAC induction of anti-tumor T cell responses. In the tumor microenvironment, the promoter region of CD80 gene in cancer cells is highly methylated, therefore CD80 is not expressed. Lack of co-stimulation signal will result in T cell tolerance. DAC treatment leads to demethylation of the promoter region of CD80 gene in cancer cells, leading to the expression of CD80, which stimulates T cell responses (Figure 9). Given the fact that DAC has already made an impact in patients with hematological malignancies and solid tumors such as lung cancer, treatment of many cancers with transient, low dose DAC or other demethylating agents may result in the desired immune response that lead to tumor rejection in these patients.

## Supporting Information

Figure S1 **Gene expression profiling of DAC-treated vs PBS-treated EL4 cells.** The mouse SmartArray chips were hybridized with RNA derived from DAC or PBS treated EL4 cells. (A) Scatter plot comparing global gene expression profiles between the DAC treated EL4 and PBS treated EL4 cells. (B) SAM plot indicating differentially expressed genes between DAC treated and PBS-treated EL4 cells. 847 genes were up-regulated by DAC.
(TIFF)
