# Supplementary figures and images for: Low Dose Decitabine Treatment Induces CD80 Expression in Cancer Cells and Stimulates Tumor Specific Cytotoxic T Lymphocyte Responses
Source: PLoS One. 2013 May 9;8(5):e62924. doi: 10.1371/journal.pone.0062924 (PMC3650049; doi:10.1371/journal.pone.0062924)

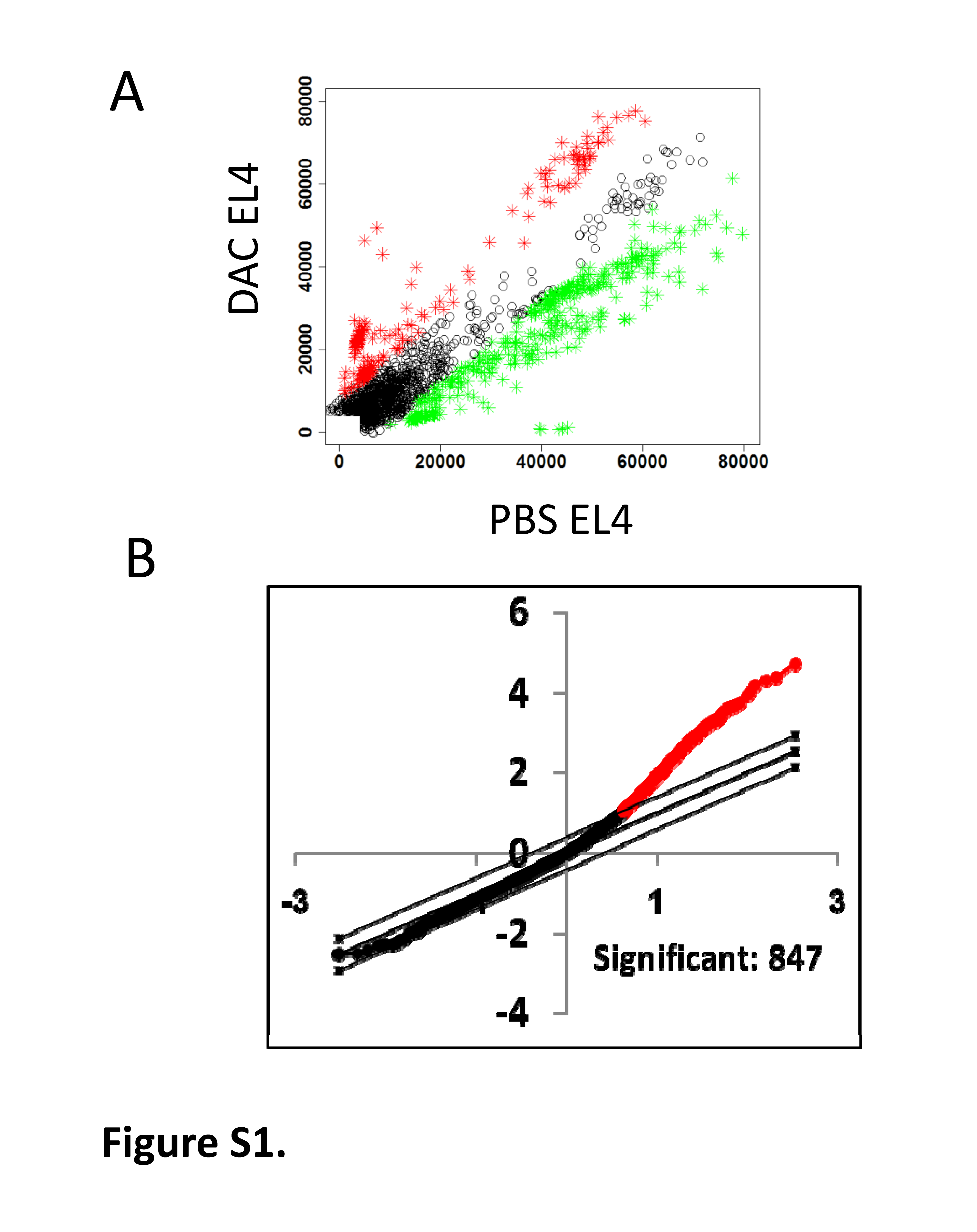

Supplement: Figure S1 — Gene expression profiling of DAC-treated vs PBS-treated EL4 cells. The mouse SmartArray chips were hybridized with RNA derived from DAC or PBS treated EL4 cells. (A) Scatter plot comparing global gene expression profiles between the DAC treated EL4 and PBS treated EL4 cells. (B) SAM plot indicating differentially expressed genes between DAC treated and PBS-treated EL4 cells. 847 genes were up-regulated by DAC. (TIFF) [file pone.0062924.s001.tiff]
